# LPS causes pericyte loss and microvascular dysfunction via disruption of Sirt3/angiopoietins/Tie-2 and HIF-2α/Notch3 pathways

**DOI:** 10.1038/srep20931

**Published:** 2016-02-12

**Authors:** Heng Zeng, Xiaochen He, Qin-hui Tuo, Duan-fang Liao, Guo-qiang Zhang, Jian-xiong Chen

**Affiliations:** 1 Department of Pharmacology and Toxicology, University of Mississippi Medical Center, School of Medicine, Jackson, MS, 39216, USA; 2 School of Integrated Chinese and Western Medicine, Hunan University of Chinese Medicine, Changsha, Hunan, 410208, China; 3 Emergency Department of China-Japan Friendship Hospital, Beijing, 100029, China

## Abstract

Recent studies reveal a crucial role of pericyte loss in sepsis-associated microvascular dysfunction. Sirtuin 3 (SIRT3) mediates histone protein post-translational modification related to aging and ischemic disease. This study investigated the involvement of SIRT3 in LPS-induced pericyte loss and microvascular dysfunction. Mice were exposed to LPS, expression of Sirt3, HIF-2α, Notch3 and angiopoietins/Tie-2, pericyte/endothelial (EC) coverage and vascular permeability were assessed. Mice treated with LPS significantly reduced the expression of SIRT3, HIF-2α and Notch3 in the lung. Furthermore, exposure to LPS increased Ang-2 while inhibited Ang-1/Tie-2 expression with a reduced pericyte/EC coverage. Intriguingly, knockout of Sirt3 upregulated Ang-2, but downregulated Tie-2 and HIF-2α/Notch3 expression which resulted in a dramatic reduction of pericyte/EC coverage and exacerbation of LPS-induced vascular leakage. Conversely, overexpression of Sirt3 reduced Ang-2 expression and increased Ang-1/Tie-2 and HIF-2α/Notch3 expression in the LPS treated mice. Overexpression of Sirt3 further prevented LPS-induced pericyte loss and vascular leakage. This was accompanied by a significant reduction of the mortality rate. Specific knockout of prolyl hydroxylase-2 (PHD2) increased HIF-2α/Notch3 expression, improved pericyte/EC coverage and reduced the mortality rate in the LPS-treated mice. Our study demonstrates the importance of SIRT3 in preserving vascular integrity by targeting pericytes in the setting of LPS-induced sepsis.

Sepsis is a major health care problem, affecting millions of individuals around the world each year. Severe sepsis and septic shock are characterized by microvascular dysfunction and vascular leakage. The endothelium controls vascular tone and permeability, regulates cellular oxygen and nutrient trafficking, and maintains microvascular homeostasis. Disruption of endothelial barrier function results in vascular destabilization and microvascular leakage which play important mechanistic role in the pathogenesis of end-organ dysfunction and septic shock^1^,^2^,^3^. Most recent studies also revealed a critical role of capillary pericytes in the sepsis-associated vascular leakage. Sepsis may cause microvascular hyper-permeability via disruption of pericyte/endothelial cell (EC) interactions^4^,^5^,^6^. Although the disruption of pericyte/EC interactions and vascular integrity is prevalent in sepsis, the underlying molecular mechanism(s) remains unexplored.

Tie-2 is an endothelial-specific receptor tyrosine kinase that is predominantly expressed in the vascular endothelium. Angiopoietin-1 (Ang-1) (agonist) and angiopoietin-2 (antagonist) are the two ligands of the Tie-2 receptor. Angiopoietins/Tie-2 signaling is essential for the maintenance of EC/pericyte integrity and vessel stability. Ang-2, a protein secreted by stimulated endothelium and an antagonist of Ang-1 and Tie-2, contributes to the pathophysiology of septic multiple organ dysfunction. Blockade of Ang-2 reversed LPS-induced hypotension and reduced the mortality rate in mice^6^. In contrast, activation of Ang-1/Tie-2 pathway increases EC/pericyte interactions and prevents vascular leakage^7^,^8^,^9^,^10^. Interestingly, Ang-1 expression was significantly reduced in sepsis^11^,^12^. Moreover, Ang-1 treatment significantly improves cardiac function and reduces lung injury in endotoxemic mice^13^,^14^,^15^,^16^,^17^,^18^,^19^; highlighting a potential causative role of imbalanced angiopoietins/Tie-2 system in sepsis. So far, the molecular mechanism(s) that leads to disruption of angiopoietins/Tie-2 system and loss of capillary pericytes in sepsis is not clearly defined.

Sirtuins (Sirtuin 1–7) belong to a highly conserved family of histone/protein deacetylases. Sirtuins mediate histone protein post-translational modification by coupling lysine deacetylation to NAD^+^ hydrolysis. Emerging evidence suggests that sirtuins play crucial role in the regulation of cell function. Sirt3 is a longevity factor that couples energy and oxygen homeostasis^20^,^21^,^22^. Our study showed that loss of Sirt3 significantly blunted apelin-mediated angiogenesis^23^. Apelin has been shown to maintain vascular integrity and attenuate vascular hyperpermeability^24^,^25^. Ang-1/Tie-2 acts as downstream mediator of apelin signaling and modulates apelin-induced angiogenesis^26^,^27^,^28^. Our recent study also showed that Sirt3 deficiency reduced HIF-2α expression and decreased pericyte number in the heart^29^. HIF-2α has been shown to be destabilized by prolyl hydroxylase-2 (PHD2). PHD2-HIF-2α axis has been reported to regulate the shape and stability of microvasculature^30^,^31^,^32^. Based upon these findings, we hypothesize that LPS causes acute pericyte loss and microvascular dysfunction by the mechanism(s) involving disruption of Sirt3/angiopoietins/Tie-2 and HIF-2α/Notch3 signaling pathways.

Using loss- and gain- of function approaches and endotoxin (LPS)-induced septic model, we have investigated the fundamental role of Sirt3 in the regulation of endothelial/pericyte interactions, vascular integrity and permeability during sepsis. Our data demonstrated that Sirt3 was necessary for the pericyte function and vascular permeability in sepsis. We further revealed that disruption of angiopoietins/Tie-2 and HIF-2α/Notch3 signaling pathways may be responsible for LPS-induced impairment of pericyte/EC coverage and microvascular dysfunction.

## Results

### LPS reduces Sirt3 levels in the lung and mouse lung endothelial cell (MLEC)

**Figure 1:**
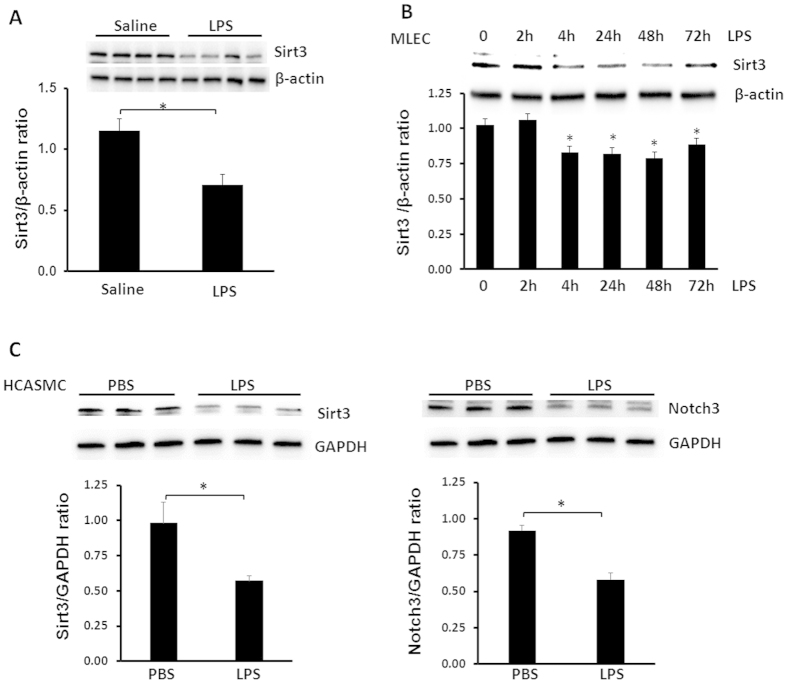
Effects of LPS on Sirt3 levels in the lung and MLEC. (**A**)Sirt3 expression was significantly reduced in the lung of WT mice at 12 hours after LPS challenge as compared to WT control mice without LPS. (n = 4 mice,*p < 0.05). (**B**) Western blot analysis showing that exposure of MLEC to LPS (10 μg/ml) led to a gradual decrease in Sirt3 expression seen at 4 hours and remained lower levels for 72 hours (n = 3,*p < 0.05). (**C**) Western blot analysis showing that exposure of HCASMC to LPS (10 μg/ml) for 24 hours resulted in a significant decrease in Sirt3 and Notch3 expression (n = 3, *p < 0.05).

To determine whether endotoxin altered Sirt3 levels *in vivo*, mice were challenged with LPS (2 mg/kg, i.p.). After 12 hours of LPS treatment, Sirt3 expression in the lung was measured by western blot analysis. As shown in Fig. 1A, Sirt3 expression was significantly reduced in the LPS treated mice compared to mice without LPS treatment. Furthermore, exposure of mouse lung endothelial cell (MLEC) to LPS (10 μg/ml) for various time up to 72 hours led to a gradual decrease in Sirt3 expression, seen at 4 hours and remained low up to 72 hours (Fig. 1B). In addition, exposure of human coronary artery smooth muscle cell (HCASMC) to LPS (10 μg/ml) for 24 hours significantly reduced Sirt3 and Notch3 expression (Fig. 1C).

### LPS increases Ang-2 and reduces Ang-1/Tie-2 expression in the lung

**Figure 2:**
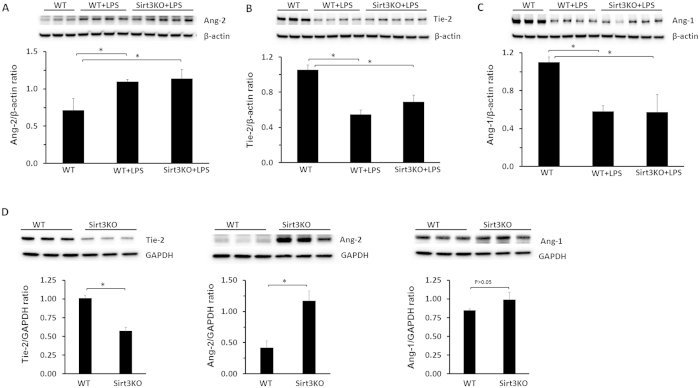
Effects of LPS on angiopoietins/Tie-2 system in the lung. (**A**) Western blot analysis demonstrating that treatment of mice with LPS for 12 hours resulted in a significant increase in Ang-2 expression in WT and Sirt3KO mice (*p < 0.05, n = 3–5 mice). (**B**) Western blot analysis showing that treatment of mice with LPS for 12 hours significantly decrease Tie-2 expression in WT and Sirt3KO mice (*p < 0.05, n = 3–5 mice). (**C**) Western blot analysis revealing that treatment of mice with LPS for 12 hours led to a significant reduction of Ang-1 expression in WT and Sirt3KO mice (*p < 0.05, n = 3–5 mice). (**D**) Western blot analysis showing that the expression of Tie-2 was downregulated whereas Ang-2 was upregulated in Sirt3KO mice compared to WT mice. The expression of Ang-1 remained unchanged (n = 3 mice, *p < 0.05).

Since angiopoietins/Tie-2 mediated pericyte function and vascular permeability, we therefore examined whether LPS affected angiopoietins and Tie-2 expression in WT and Sirt3KO mice. In WT mice, treatment of mice with LPS for 12 hours resulted in a significant increase in Ang-2 expression (Fig. 2A). This was accompanied by a significant reduction of Tie-2 and Ang-1 expression in the lung (Fig. 2B,C). Under unstimulated conditions, knockout of Sirt3 caused a significant decrease in Tie-2 and increase in Ang-2 expression in the lung as compared to WT mice (Fig. 2D). Under LPS stimulated conditions, however, knockout of Sirt3 in mice failed to further upregulate Ang-2 and downregulate Ang-1/Tie-2 expression (Fig. 2A–C).

### Overexpression of Sirt3 increases Ang-1/Tie-2 and reduces Ang-2 expression after LPS challenge

**Figure 3:**
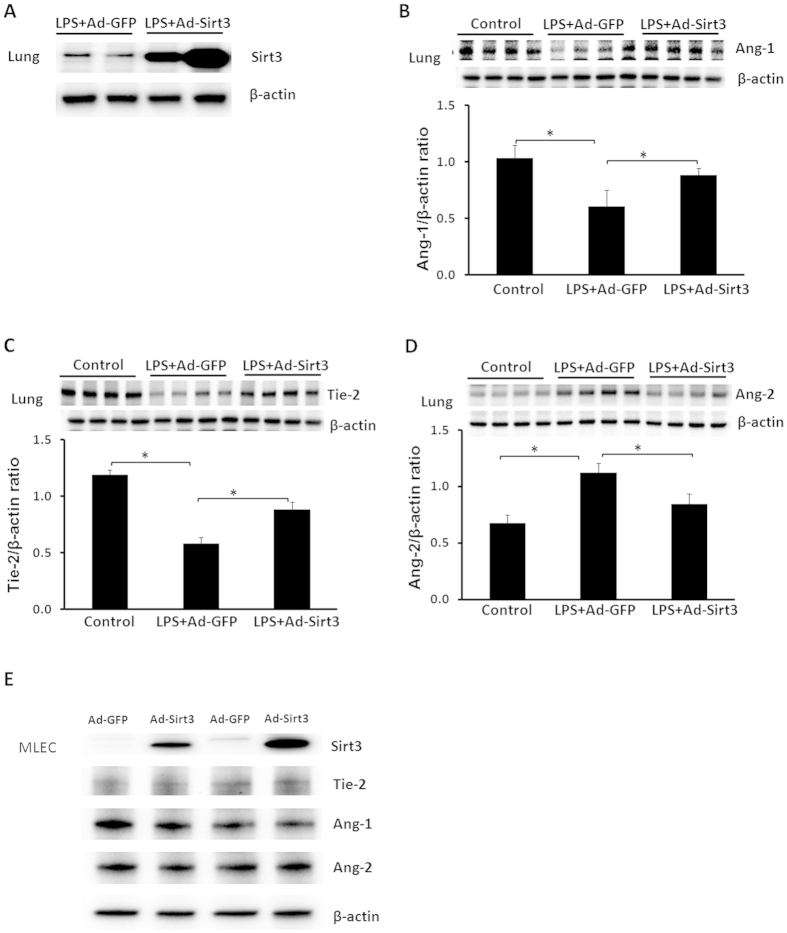
Overexpression of Sirt3 reversed LPS-induced angiopoietins/Tie-2 abnormality in the lung. (**A**) Sirt3 levels were dramatic increased in the lung of WT+Ad-Sirt3 treated mice at 12 hours after LPS challenge as compared to WT + LPS mice with Ad-GFP treatment. (**B**) Treatment of LPS mice with Ad-Sirt3 significantly increased Ang-1 expression in the lung as compared to WT + LPS mice with Ad-GFP treatment. n = 4 mice,*p < 0.05. (**C**) Treatment of LPS mice with Ad-Sirt3 significantly increased Tie-2 expression in the lung as compared to WT + LPS mice with Ad-GFP treatment. *p < 0.05, n = 4 mice. (**D**) Western blot analysis showing that treatment of mice with Ad-Sirt3 significantly blunted LPS-induced Ang-2 expression in the lung as compared WT + LPS mice with Ad-GFP treatment. *p < 0.05, n = 4 mice. (**E**) Western blot analysis showing that MLMC transfected with Ad-Sirt3 increased Sirt3 expression, but did not alter basal levels of Ang-1, Ang-2 and Tie-2 expression.

To further test whether LPS-induced reduction of Sirt3 contributed to imbalanced angiopoietins/Tie-2 system, mice were pretreated with Ad-Sirt3 before challenged with LPS. In this study, Ad-Sirt3 (1 × 10^7^ PFU) or Ad-GFP (1 × 10^7^ PFU) was intravenously injected into the experimental mice. After 48 hours, experimental mice were injected with LPS (2 mg/kg i.p.). Systemic administration of Ad-Sirt3 resulted in a dramatic increase in Sirt3 levels in the lung of LPS treated mice (Fig. 3A). Moreover, Ad-Sirt3 treatment led to a significant increase in Ang-1 and Tie-2 expression in LPS-treated mice (Fig. 3B,C). In contrast, Ang-2 expression was reduced in Ad-Sirt3 + LPS mice (Fig. 3D). Transfection of MLEC with Ad-Sirt3 increased Sirt3 expression, but did not alter the basal levels of Ang-1, Ang-2 and Tie-2 expression (Fig. 3E).

### Sirt3 ablation exacerbates LPS-induced vascular leakage and cardiac dysfunction in mice

**Figure 4:**
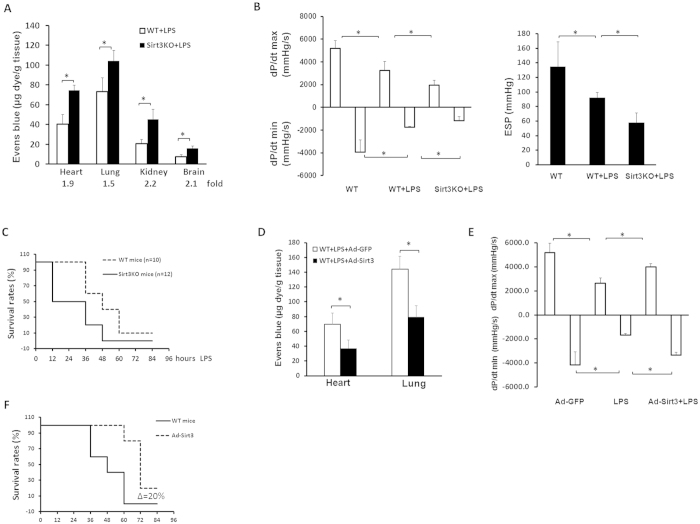
Effects of Sirt3 ablation on LPS-induced vascular leakage and cardiac dysfunction. (**A**) Quantitative analysis of vascular permeability using Evans blue dye and expressed as ng of dye per mg of tissue. Vascular permeability was significantly increased in the heart, lung, kidney and brain of Sirt3KO mice + LPS when compared with WT mice + LPS. *p < 0.05, n = 7–10 mice. (**B**) L-V loop analysis showing that cardiac function was depressed in WT and Sirt3KO mice after LPS treatment. Knockout of Sirt3 significantly enhanced LPS-induced suppression of cardiac function compared to WT + LPS (n = 4–5 mice,*p < 0.05). (**C**) Ablation of Sirt3 increased the mortality rate after lethal LPS challenge (n = 10–12 mice,*p < 0.05). (**D**) Vascular permeability measured by Evans blue dye was significantly reduced in the heart and lung of Ad-Sirt3 + LPS mice when compared with Ad-GFP +  LPS mice. *p < 0.05, n = 7 mice. (**E**) Cardiac function was reduced in WT mice at 12 hours after LPS treatment as compared to WT control mice without LPS. Ad-Sirt3 treatment significantly attenuated LPS-induced cardiac dysfunction compared to WT + LPS treatment (n = 5 mice,*p < 0.05). (**F**) Overexpression of Sirt3 significantly reduced the mortality rate after lethal dose LPS (20 mg/kg) challenge in mice (n = 10, *p < 0.05).

Using an Evens Blue assay, we measured whether loss of Sirt3 altered vascular permeability in the heart, lung, kidney and brain after challenge with LPS for 24 hours. Knockout of Sirt3 resulted in a significant increase in vascular permeability in these organs when compared with control WT mice treated with LPS (Fig. 4A). Knockout of Sirt3 did not alter basal vascular permeability under the unstimulated conditions (data not shown). The tail cuff measurement of MAP also showed that the basal levels of MAP were similar between WT and Sirt3KO mice (WT MAP = 103.5 ± 2.7 mmHg vs Sirt3KO MAP = 102.7 ± 3.5 mmHg, n = 6 mice, p > 0.05). Cardiac function was measured by P-V loop at 12 hours after LPS injection. The dp/dt max and end systolic pressure (ESP) were significantly reduced whereas dp/dt min was increased in Sirt3KO mice +LPS compared to WT mice + LPS (Fig. 4B). In addition, the mortality rate was greater in Sirt3KO mice than WT mice. All Sirt3KO mice died within 48 hours after lethal dose LPS (20 mg/kg) challenge, but WT mice had about 40% survival rate within 48 hours after LPS challenge (Fig. 4C).

### Overexpression of Sirt3 attenuates LPS-induced vascular leakage and reduces the mortality rate in mice

Next, we examined whether overexpression of Sirt3 could rescue LPS-induced vascular leakage and reduce the mortality rate in mice. Treatment of mice with Ad-Sirt3 resulted in a significant decrease in vascular permeability in the heart and lung when compared with control mice treated with LPS (Fig. 4D). As shown in Fig. 4E, treatment with Ad-Sirt3 further led to a significant improvement of cardiac function when compared to WT mice challenged with LPS. Ad-Sirt3 treatment significantly reduced the mortality rate after lethal dose LPS (20 mg/kg) challenge in mice (Fig. 4F).

### Sirt3 regulates LPS-induced pericyte loss and inflammatory infiltration

**Figure 5:**
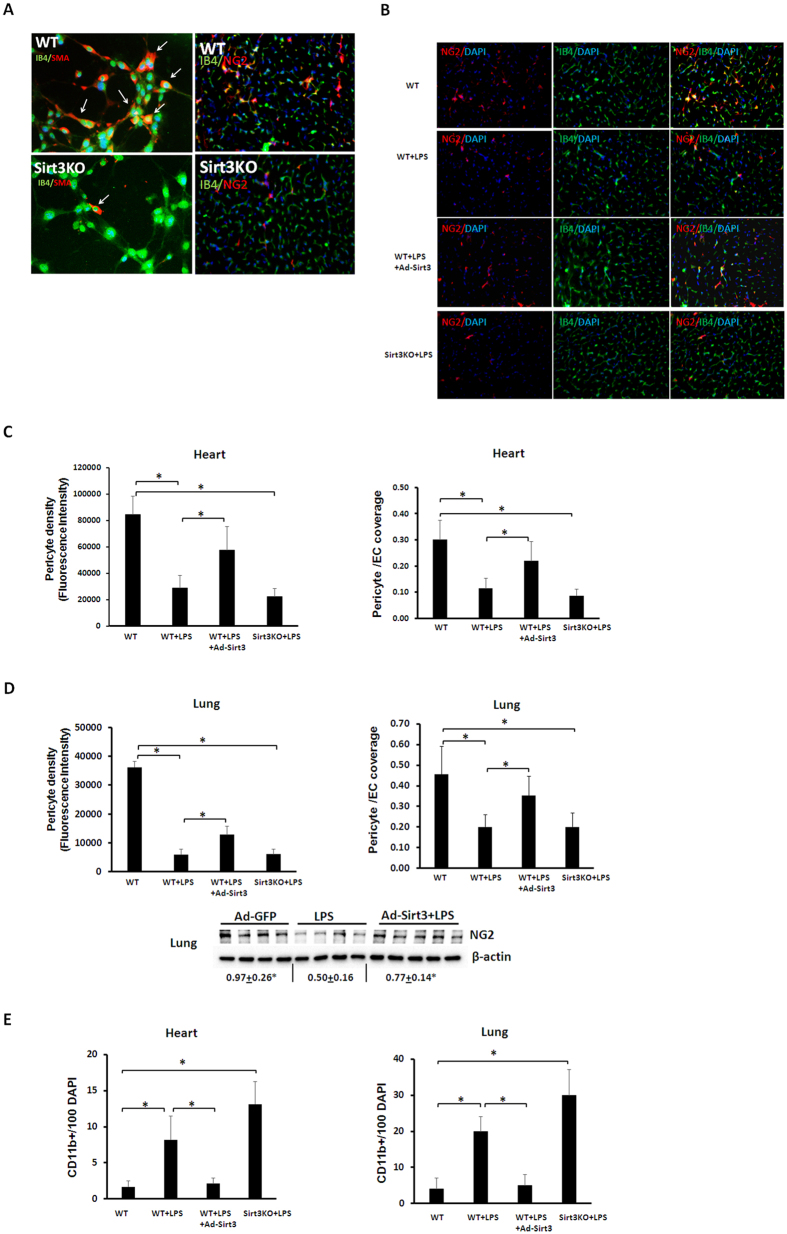
Loss and gain of Sirt3 on LPS-induced pericyte loss and macrophage infiltration. (**A**) Left panel: Representative images of EC/pericyte coverage (white arrow) in *ex vivo* model of vessel explants isolated from WT and Sirt3KO mice. Sirt3KO mice had few EC/pericyte coverage. EC was stained with EC marker IB4 (green, 20x) and pericytes were stained by SMA (Red, 20x). Right panel: Sirt3KO mice displayed loss of capillary associated pericytes (red) in the heart. ECs were stained with EC marker IB4 (green, 40x) and pericytes were stained by NG2 (Red, 40x). (**B**) Representing images of pericyte density and pericyte/EC coverage in the heart of LPS treated mice. NG2 (red), IB4 (green) and DAPI (blue), 40X. (**C**) Quantification of pericyte/EC coverage determined as ratio of NG2 (red) to Isolectin-B4 (IB4, green) staining and pericyte density measured as relative density of red fluorescence in the heart (n = 6 mice,*p < 0.05). (**D**) Quantification of pericyte density and pericyte/EC coverage in the lung tissue (n = 6 mice,*p < 0.05). Western blot analysis confirming that exposure to LPS caused a dramatic decrease in NG2 expression whereas overexpression of Sirt3 rescued LPS-induced downregulation of NG2 in the lung. (**E**) Quantitative analysis of macrophage infiltrations (CD11b positive cells) showing that the number of macrophage infiltration was increased in the heart and lung of WT and Sirt3KO mice after challenged with LPS compared to control with LPS treatment. Overexpression of Sirt3 dramatic reduced the number of macrophage in the heart and lung of LPS treated mice (n = 6 mice,*p < 0.05).

Using loss- and gain- of function approaches, we examined direct role of Sirt3 on LPS-induced pericyte loss and impairment of pericyte/EC coverage. Under basal conditions, knockout of Sirt3 resulted in a dramatic reduction of pericyte/EC coverage in vessel explants and in the heart (Fig. 5A). WT mice challenged with LPS for 12 hours resulted in acute pericyte loss with a reduction of pericyte/EC coverage in the heart (Fig. 5B,C). Similarly, pericyte density and pericyte/EC coverage were significantly reduced in the lung of LPS treated mice (Fig. 5D). Western blot analysis also confirmed a dramatic reduction of NG2 protein levels in the lung after LPS challenge (Fig. 5D). This was accompanied by a significant increase in neutrophil/macrophage (CD11b^+^) infiltration both in the heart and lung (Fig. 5E). Knockout of Sirt3 did not further reduce pericyte, but significantly exacerbated neutrophil/macrophage (CD11b^+^) infiltration as compared to WT mice treated with LPS (Fig. 5E). In contrast, overexpression Sirt3 increased pericyte density and improved pericyte/EC coverage in the LPS treated mice (Fig. 5B–D). Overexpression of Sirt3 also increased pericyte NG2 expression in the lung of LPS treated mice (Fig. 5D). Moreover, overexpression of Sirt3 led to a significant reduction of CD11b^+^ accumulation in the heart and lung after LPS challenge (Fig. 5E). Under unstimulated conditions, overexpression of Sirt3 had little effects on pericyte/EC coverage and CD11b^+^ accumulation in the heart (data not shown).

### Knockout of PHD2 increases HIF-2α/Notch3 and attenuates LPS-induced pericyte loss

**Figure 6:**
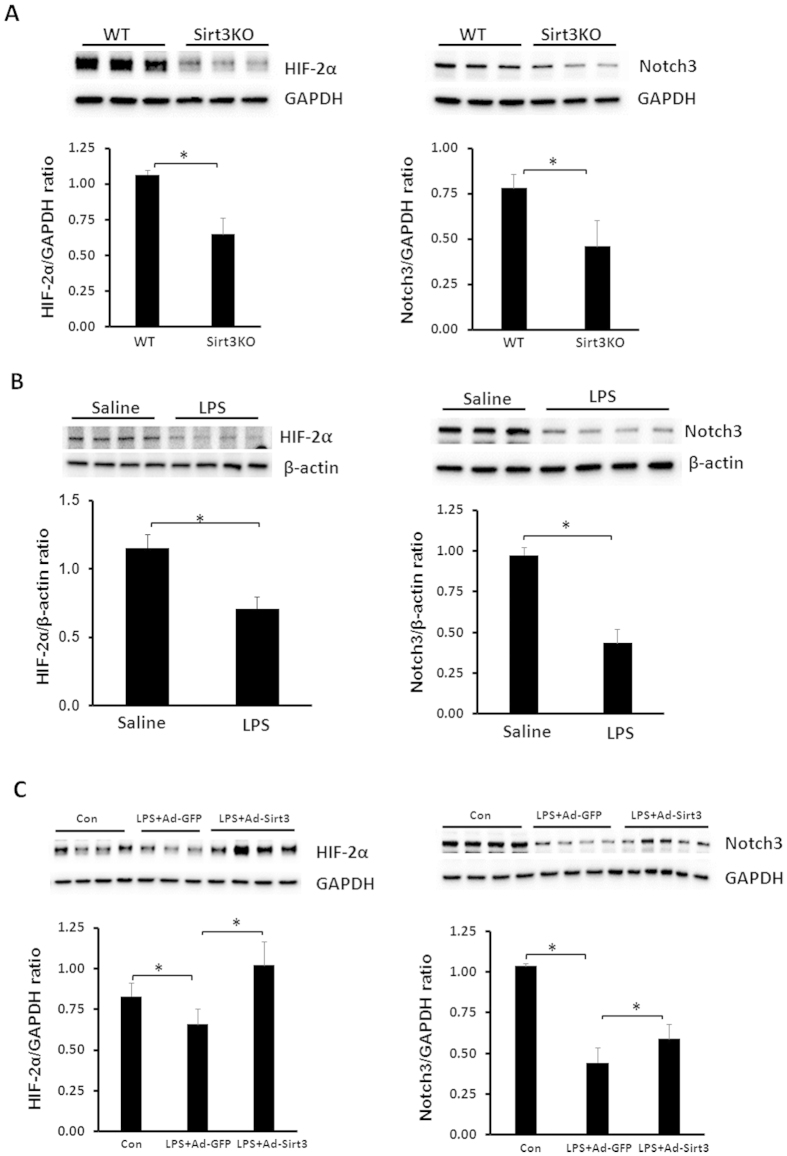
Effects of LPS and Sirt3 on HIF-2α and Notch3 expression. (**A**)Western blot analysis revealing that knockout of Sirt3 reduced HIF-2α and Notch3 expression in the lung compared to WT control mice (*p < 0.05, n = 3 mice). (**B**) Western blot analysis demonstrating that exposure of WT mice to LPS resulted in a significant decrease in HIF-2α and Notch3 expression compared to WT control mice (*p < 0.05, n = 3–4 mice). (**C**) Western blot analysis showing that overexpression of Sirt3 significantly increased HIF-2α and Notch3 expression compared to WT control mice +LPS (*p < 0.05, n = 3–5 mice).

**Figure 7:**
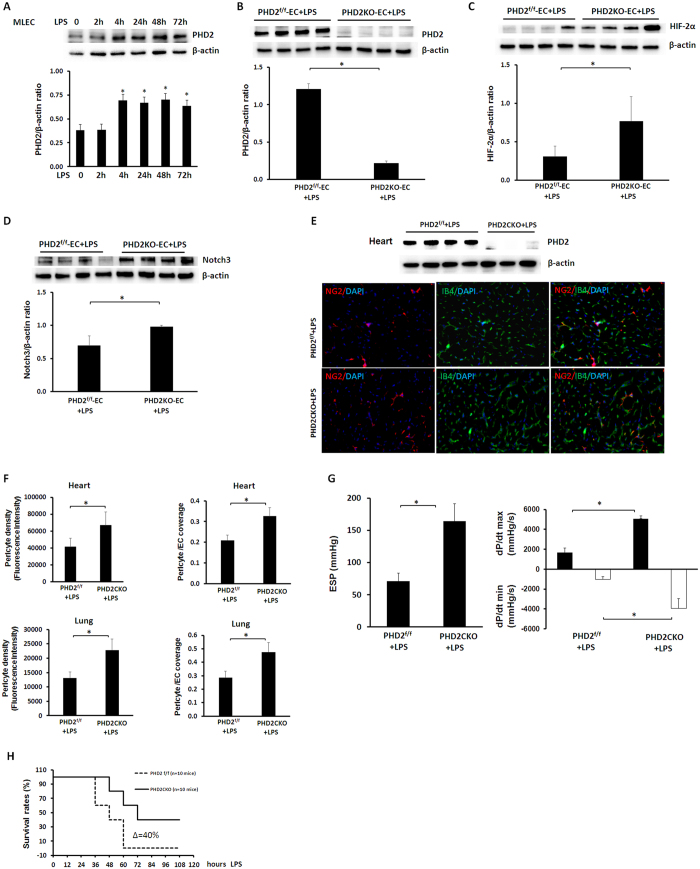
Effects of PHD2 ablation on LPS-induced pericyte loss and cardiac dysfunction. (**A**) Exposure of MLEC to LPS for 72 hours resulted in a gradual increase in endothelial PHD2 expression (n = 4 mice,*p < 0.05). (**B**) Western blot analysis showing that PHD2 levels were significantly reduced in the MLEC isolated from PHD2 conditional knockout mice (n = 4 mice,*p < 0.05). (**C**) Western blot analysis demonstrating that deletion of PHD2 in MLEC resulted in a significant increase in HIF-2α expression in LPS treated MLEC (n = 4 mice,*p < 0.05). (**D**) Western blot analysis demonstrating that deletion of PHD2 in MLEC significantly increase Notch3 expression in LPS treated MLEC (n = 4 mice,*p < 0.05). (**E**) Western blot analysis confirming that PHD2 gene was deleted in the heart of PHD2 conditional knockout mice challenged with LPS. Representing images of pericyte density and pericyte/EC coverage in the heart of PHD2^f/f^ + LPS mice and PHD2CKO + LPS mice. NG2 (red), IB4 (green) and DAPI (blue), 40X. (**F**) Quantitative analysis of pericyte/EC coverage and pericyte density revealing that conditional knockout of PHD2 in LPS treated mice significantly increased pericyte density and pericyte/EC coverage in the heart and lung when compared with PHD2^f/f^ + LPS treated mice (n = 6 mice,*p < 0.05). (**G**) Conditional knockout of PHD2 in LPS treated mice led to a significant improvement of cardiac function when compared with PHD2^f/f^ + LPS treated mice (n = 10 mice,*p < 0.05). (**H**) Conditional of PHD2 resulted in approximately 40% increase in mouse survival rate after LPS challenge compared to PHD2^f/f^ + LPS treated mice (n = 10 mice,*p < 0.05).

Under unstimulated conditions, knockout of Sirt3 led to a significant reduction of HIF-2α and Notch3 expression in the lung (Fig. 6A). Similarly, exposure to LPS resulted in a dramatic reduction of HIF-2α and Notch3 expression in the lung (Fig. 6B). In contrast, overexpression of Sirt3 increased HIF-2α and Notch3 expression in LPS-treated mice (Fig. 6C). Exposure of MLEC to LPS also caused a significant increase in PHD2 expression (Fig. 7A). Knockout of PHD2 in MLEC was confirmed by western blot analysis. Deletion of PHD2 in MLEC resulted in a significant increase in HIF-2α expression after exposure to LPS (Fig. 7B,C). Interestingly, deletion of PHD2 significantly increased Notch3 expression in MLEC exposed to LPS (Fig. 7D). Using PHD2 conditional knockout mice, we further examined whether deletion of PHD2 attenuated LPS-induced pericyte loss. As shown in Fig. 7E,F, conditional knockout of PHD2 led to a significant increase in pericytes and pericytes/EC coverage in LPS treated mice. Moreover, conditional knockout of PHD2 significantly enhanced cardiac function and improved mouse survival rate after lethal dose LPS (20 mg/kg) challenge (Fig. 7G,H).

## Discussion

In the present study, we have demonstrated that reduction of Sirt3 was a critical factor for LPS-induced pericyte loss and microvascular dysfunction in mice. Our data showed that Sirt3 levels were significantly reduced in the lung and MLEC exposed to LPS. This was accompanied by a significant increase in Ang-2 and decreases in Ang-1/Tie-2 and HIF-2α/Notch3 expression. Intriguingly, knockout of Sirt3 in mice led to a significant increase in Ang-2 and decreases in Tie-2 and HIF-2α/Notch3 expression. Knockout of Sirt3 also exhibited a significant reduction of pericyte and pericyte/EC coverage. These abnormalities may contribute to the exacerbation of LPS-induced microvascular permeability. Conversely, overexpression of Sirt3 reversed LPS-induced pericyte loss, attenuated vascular permeability and neutrophil/macrophage infiltration and reduced the mortality rate in LPS treated mice. Mechanistically, overexpression of Sirt3 increased Ang-1/Tie-2 and HIF-2α/Notch3, and suppressed Ang-2 expression in LPS treated mice. Furthermore, specific knockout of PHD2 increased HIF-2α/Notch3 expression and attenuated LPS-induced pericyte loss. Our data suggest that LPS may cause pericyte loss and microvascular dysfunction by a mechanism involving disruption of Sirt3/angiopoietins/Tie-2 and HIF-2α/Notch3 signaling pathways.

Sepsis is characterized by profound hemodynamic alterations associated with microcirculatory dysfunction. Multiple experimental studies have found that sepsis induces capillary collapse and results in a marked decrease in capillary blood flow^33^,^34^. Pericytes are vascular mural cells of mesenchymal origin, embedded in the basement membrane of microvasculature, where they make specific local contacts with endothelium^35^,^36^. For a long time, pericytes were thought to solely maintain capillary tone. However, recent genetic studies involving mouse mutants with reduced pericyte coverage of blood vessels indicate that pericytes actually have multiple effects on the vasculature. Pericytes participate in physiological and pathological vascular stability and permeability. It has been shown that vascular permeability is controlled by pericytes in the blood-brain barrier (BBB). Cerebral vascular permeability is significantly increased with time from 1–24 hour(s) after LPS treatment. Intriguingly, this impairment of BBB function is associated with detachment of pericytes from endothelial cells^37^. Loss of pericytes also leads to an increase in microvascular permeability in mice^38^. Moreover, mice challenged with LPS results in acute pericyte loss and vascular hyperpermeability in the heart^6^. Consistent with these findings, we found that mice challenged with LPS led to a significant loss of pericytes in the microvasculature. This was accompanied by a dramatic increase in vascular leakage and neutrophil/macrophage infiltration. To further determine whether pericyte apoptosis contributed LPS-induced pericyte loss in the microvasculature, NG2 positive cells were co-stained with apoptotic marker TUNEL. There were no co-stained NG2^+^ cells with TUNEL (data not shown), indicating that LPS-induced pericyte loss may be due to the detachment from endothelium. Despite the known benefit of preventing pericyte loss and maintaining EC/pericyte integrity, little is known about the intracellular mechanisms underlying loss of pericytes and disruption of EC/pericyte interactions in sepsis. Our present study showed that Sirt3 levels were reduced after challenged with LPS. Moreover, knockout of Sirt3 exacerbated LPS-induced vascular leakage. In contrast, overexpression of Sirt3 reversed LPS-induced pericyte loss and improved pericyte/EC coverage together with improvement of vascular stability. Our present data also showed that exposure to LPS caused a significant capillary loss (data not shown). Surprisingly, knockout of Sirt3 or overexpression of Sirt3 had little effect on LPS-induced loss of capillaries, suggesting that Sirt3 may specific target pericytes in the setting of sepsis and septic shock. To our knowledge, this is the first study demonstrated that a reduction of Sirt3 contributes to pericyte loss and microvascular dysfunction in sepsis.

Angiopoietins, PDGFB and S1P play crucial roles in the regulation of pericyte functions and microvascular integrity. Therapeutic targeting of these molecules and signaling pathways have been shown to prevent or limit end-organ dysfunction and injury in sepsis^6^,^16^,^17^,^18^,^39^,^40^,^41^. Increased Ang-2 levels in septic individuals are strongly associated with severity of illness and adverse outcome. Overexpression of Ang-2 in EC, but not in cardiomyocyte, causes sepsis-like hemodynamic alterations including systemic hypotension, increased vascular permeability and dilatory cardiomyopathy with pericyte loss^6^. Ang-2-induced hemodynamic alterations and loss of capillary-associated pericytes can be rescued by overexpression of Ang-1 and PDGFB^6^. These data suggest that stabilization of pericyte attachment to the capillary network is a critical step in preventing vascular leakage and hemodynamic dysregulation in sepsis. In keeping with these, our study showed that Ang-2 levels were increased together with a significant pericyte loss in the LPS treated mice and Sirt3KO mice. Exposure to LPS caused a significant reduction of Ang-1/Tie-2 expression. Our present data also showed that knockout of Sirt3 did not further reduce LPS-induced downregulation of Ang-1/Tie-2 expression. Therefore, the Sirt3 deficiency-induced imbalanced angiopoietin/Tie-2 system and impaired pericyte/EC coverage under basal conditions may be attributed to the exacerbation of LPS-induced vascular leakage. Using loss- and gain-function approaches, our present study demonstrated a causative relationship between reduction of Sirt3 levels and disruption of angiopoietins/Tie-2 system in the setting of LPS-induced sepsis. Overall, our present study provides direct evidence that LPS-induced reduction of Sirt3 may mediate pericyte loss and microvascular leakage via angiopoietins/Tie-2 system.

In the present study, we found that exposure to LPS caused a significant reduction of HIF-2α and Notch3 expression in the lung. We also found that overexpression of Sirt3 upregulated HIF-2α and Notch3 expression in the LPS treated mice. Most interestingly, exposure to LPS led to a significant increase in PHD2 levels in EC. Endothelial cells in “quiescent” state display a “phalanx cell” phenotype in which ECs are aligned in a tight cobblestone pattern. The uniformity of the EC “phalanx” phenotype is regulated by PHD2-HIF-2α signaling. Reduction of PHD2 by heterozygosity promotes a phalanx-like phenotype in EC, and enhances vascular integrity in tumor vasculature via upregulation of HIF-2α/VE-cadherin. Inhibition of PHD2 also increases EC/pericyte coverage and basement membrane stabilization^30^. These data highlight the important role of PHD2 on vascular morphogenesis and permeability. PHD2 serves as an oxygen sensor and regulates oxygen delivery via modulation of vessel morphogenesis and vascular maturation. Inhibition of PHD2 increases EC/pericyte interactions and promotes vascular stabilization^42^. Furthermore, haplodeficiency of PHD2 in EC increases tissue oxygenation by normalizing the endothelial lining and forming a “phalanx” EC phenotype^30^. Our previous study showed that overexpression of Ang-1 maintained vascular integrity and stability via suppression of PHD2 and improvement of EC/pericyte interactions in diabetes^43^. We therefore tested whether knockout of PHD2 reversed sepsis-associated EC/pericyte dysfunction and microvascular dysfunction. Using conditional PHD2 knockout mice subjected to LPS challenge, we demonstrated that ablation of PHD2 significantly improved cardiac function and reduced the mortality rate. Our data further revealed that deletion of PHD2 increased HIF-2α and Notch3 expression in EC. This was accompanied by a dramatic increase in pericyte/EC coverage. Notch3 have been shown to regulate pericyte number and vascular integrity in the brain^44^,^45^. Studies have showed that Notch3KO mice challenged with Angiotensin-II upregulated Ang-2 and promoted microvascular destabilization^46^. Our data revealed that Notch3 expression was decreased whereas Ang-2 expression was increased in the lung of Sirt3KO mice. Moreover, knockout of Notch3 in mice altered coronary microvascular phenotype (loss of cardiac pericytes) with a reduction of coronary blood flow reserve. This was accompanied by a cardiac dysfunction (Zeng H and Chen JX *et al.* unpublished 2015). Taken together, our data indicate that blockade of PHD2 may attenuate LPS-induced pericyte loss and disruption of vascular integrity via upregulation of HIF-2α/Notch3 signaling pathway.

**Figure 8:**
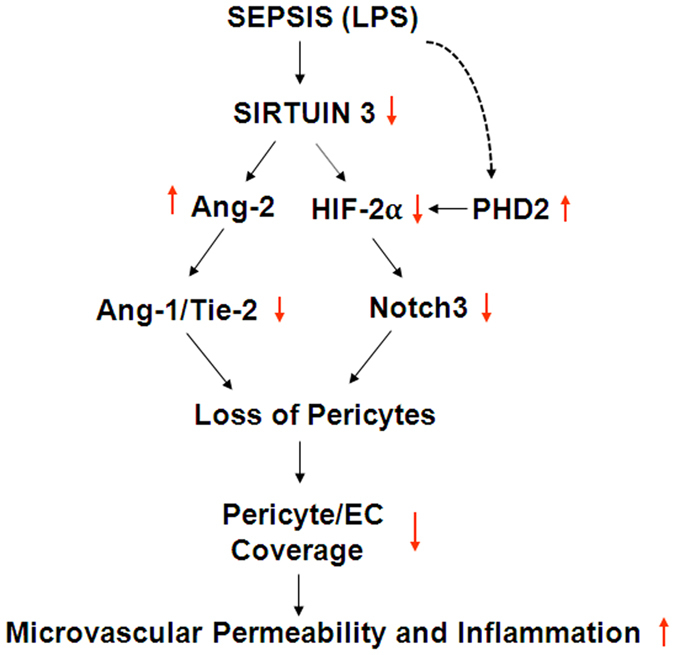
Proposed mechanisms of LPS-induced pericyte loss and microvascular dysfunction in the setting of sepsis. In endotoxemic conditions, LPS downregulates Sirt3 and upregulates PHD2 expression, thus leading to impairment of angiopoietins/Tie-2 and HIF-2α/Notch3 signaling; loss of pericytes and reduction of pericyte/capillary coverage, subsequently to promote vascular leakage, inflammation and microvascular dysfunction.

In summary, our present study revealed novel role of Sirt3 in sepsis-induced pericyte loss and microvascular dysfunction. In sepsis, LPS suppresses Sirt3 expression, which disrupts angiopoietins/Tie-2 and HIF-2α/Notch3 signaling pathways; thus leading to a reduction of pericyte/capillary coverage, subsequently promoting vascular leakage, inflammation and end-organ dysfunction (Fig. 8). We therefore proposed that SIRT3 may be potential therapeutic target for preventing sepsis-induced microvascular permeability and end-organ failure.

## Methods

All procedures conformed to the Institute for Laboratory Animal Research Guide for the Care and Use of Laboratory Animals and were approved by the University of Mississippi Medical Center Animal Care and Use Committee (Protocol ID: 1280A). The investigation conforms to the Guide for the Care and Use of Laboratory Animals published by the US National Institutes of Health (NIH Publication No. 85-23, revised 1996).

### Culture of Lung Microvascular Endothelial Cell (MLEC) and Human Coronary Artery Smooth Muscle Cells (HCASMC)

Microvascular lung endothelial cells (MLECs) were isolated from mice. In brief, mice were anesthetized with ketamine (100 mg/kg) and xylazine (15 mg/kg). The lung were perfused with 10 ml chilled PBS containing 2.5 mM EDTA, followed by perfusion of 5 ml of chilled 0.25% trypsin/2 mM EDTA through the right ventricle of the heart. The heart and lung were then removed *en bloc* and incubated at 37 °C for 20 min. Small cuts were made in the visceral plura and the ECs can be harvested by pipetting 1.5 ml EGM-2 supplemented with growth factors and 10% fetal bovine serum (FBS) up and down 10–15 times. The cell suspension was filter through a 100 μm filter and plated into 60 mm cell culture dish. After 3 days of incubation, ECs formed small colonies and non-ECs were removed by vacuum until >90% ECs left. Purity of these cells was checked by staining with rabbit anti-von Willebrand factor (vWF) polyclonal antibody (Santa Cruz biotechnology). The MLECs were cultured in EGM-2 medium at 37 °C with 5% CO_2_. Cells between passage 4 and 6 were used for all studies. MLECs were exposed to LPS (10 μg/ml) for various time up to 72 hours.

Human coronary artery smooth muscle cells (HCASMC) were purchased from Invitrogen (Carlsbad, CA). The HCASMC were cultured in medium 231 supplemented with smooth muscle differentiation supplement (Gibco Invitrogen Cell Culture, Carlsbad, CA). HCASMC at passage 4 were exposed to LPS (10 μg/ml) for 24 hours.

### LPS-induced sepsis in mice

Global Sirt3 knockout mice and wild type control of Sirt3 mice (WT) were purchased from Jackson laboratory (Bar Harbor, ME) and breeding by our laboratory. PHD2^f/f^ mice were provided by Dr. Fong at University Connecticut. The PHD2^flox/flox^ (PHD2^f/f^) mouse was crossed with B6-ROSA-Cre/ERT2 to generate a PHD2^f/f^-Cre^+^ mouse^47^. PHD2^f/f^ and PHD2^f/f^-Cre^+^ mice were bred by our colonies. Male PHD2^f/f^ and PHD2^f/f^-Cre^+^ mice at age of 8 weeks were administrated with tamoxifen (1mg/day in corn oil, Sigma, MO) for 7 days to delete PHD2 before LPS challenge. Two weeks after tamoxifen administration, mice were used for the experiments. Deletion of PHD2 gene was confirmed by western blot analysis. Experimental mice were injected with lipopolysaccharides (2 mg/kg, i.p., LPS, Sigma). Purified Ad-GFP and Ad-Sirt3 were obtained from Vector BioLabs (Malvern, PA). The experimental mice were received an intravenous jugular vein injection of Ad-GFP (1 × 10^7^ PFU) or Ad-Sirt3 (1 × 10^7^ PFU) for 48 hours before LPS challenge. For the survival studies, experimental mice were challenged with lethal dose LPS (20 mg/kg).

### Western analysis of HIF-2α, PHD2, Sirt3, Notch3, NG2, Tie-2, Ang-1 and Ang-2 expression

The lung, HCASMC and MLEC were harvested and homogenized in 300 μl of lysis buffer and total protein concentrations were determined using a BCA protein assay kit (Pierce Co, IL). Fifteen microgram of protein were subjected to SDS-PAGE on 10% polyacrylamide gels and transferred to a PVDF membrane. The blots were probed with Sirt3 and Tie-2 (1:1000, Cell Signaling, MA), NG2 (1:1000, abcam), PHD2 and HIF-2α (1:1000, Novus Bio, CO), Notch3 and Ang-2 (1:1000, Santa Cruz, CA) and Ang-1 (1:1000, Sigma, MO) antibodies. The membranes were then washed and incubated with a secondary antibody coupled to horseradish peroxidase and densitometric analysis was carried out using image acquisition and analysis software (TINA 2.0).

### Pericyte/EC coverage and neutrophil/macrophage infiltration

To assess the EC and pericyte densities, heart and lung tissues were sectioned with thickness of 10 μm. Endothelial cells were labeled with Alexa 488-labeled Isolectin B4 (IB4). Pericytes were immunolabeled with NG2 antibody (1:100, abcam). Relative density of fluorescence was quantified by measuring 6 random microscopic fields per mouse heart and lung using image-analysis software (Image J, NIH). The ratio of pericyte/endothelial density was calculated as index of pericyte/EC coverage^43^. The infiltration of neutrophil/macrophage in the heart and lung was assessed by stained with macrophage specific antibody CD11b^47^.

### Vascular permeability measurement

Vascular permeability in the heart, lung, kidney and brain was examined after 24 hours of LPS challenge. Capillary permeability was measured using an Evans-blue assay and expressed as μg dye per gram tissue^48^.

### Hemodynamic measurements

Experimental mice were anesthetized with ketamine (100 mg/kg) plus xylazine (15 mg/kg), intubated and artificially ventilated with room air. A 1.4-Fr pressure–conductance catheter (SPR-839, Millar Instrument, TX) was inserted into the left ventricle (LV) to record baseline cardiac hemodynamics of the heart. Raw conductance volumes were corrected for parallel conductance by the hypertonic saline dilution method^23^.

### Statistical analysis

The results were expressed as the mean ± SD. Statistical analysis was performed using one way ANOVA followed by multiple comparisons test (Student-Newman-Keuls) or student t-test. Significance was set at *P* < 0.05.

## Additional Information

**How to cite this article**: Zeng, H. *et al.* LPS causes pericyte loss and microvascular dysfunction via disruption of Sirt3/angiopoietins/Tie-2 and HIF-2α/Notch3 pathways. *Sci. Rep.* **6**, 20931; doi: 10.1038/srep20931 (2016).
